# Characterization of exosome-like vesicles derived from *Taenia pisiformis* cysticercus and their immunoregulatory role on macrophages

**DOI:** 10.1186/s13071-020-04186-z

**Published:** 2020-06-19

**Authors:** Li-Qun Wang, Ting-Li Liu, Pan-Hong Liang, Shao-Hua Zhang, Tao-Shan Li, Yan-Ping Li, Guang-Xue Liu, Li Mao, Xue-Nong Luo

**Affiliations:** 1 State Key Laboratory of Veterinary Etiological Biology, Key Laboratory of Veterinary Parasitology of Gansu Province, Lanzhou Veterinary Research Institute, Chinese Academy of Agricultural Sciences, Lanzhou, 730046 Gansu Province People’s Republic of China; 2 Jiangsu Co-Innovation Center for the Prevention and Control of Important Animal Infectious Disease and Zoonoses, Yangzhou University, Yangzhou, 225009 People’s Republic of China

**Keywords:** Cysticercus pisiformis, Exosome-like vesicles, Macrophages, Th2-type immune response

## Abstract

**Background:**

*Taenia pisiformis* is one of the most common intestinal parasites in canines, and leads to serious economic losses in the rabbit breeding industry. Exosome-like vesicles from parasites play crucial roles in host-parasite interactions by transferring cargo from parasites to host cells and by modulating host immunological response through inducing production of host-derived cytokines. Nevertheless, the mechanism by which exosome-like vesicles from *T. pisiformis* cysticercus regulate the macrophage immune response remains unknown.

**Methods:**

Using ultracentrifugation, we isolated exosome-like vesicles from excretory/secretory products (ESP) of *T. pisiformis* cysticercus. The morphology and size of purified vesicles were confirmed by transmission electron microscopy (TEM) and nanoparticle tracking analysis (NTA). The components of proteins and miRNAs within these vesicles were identified by proteomic analysis and high-throughput small RNA sequencing. The biological function of targets of exosomal miRNAs was predicted by Kyoto Encyclopedia of Genes and Genomes (KEGG) pathway analysis. Moreover, the expression of Th1- and Th2-type immune response associated cytokines in RAW264.7 macrophages were evaluated by qPCR and ELISA. We found that exosome-like vesicles were typical cup-shaped vesicles with diameters from 30 to 150 nm. A total of 87 proteins were identified by proteomic analysis, including proteins prominently associated with exosome-like vesicles biogenesis and vesicle trafficking. 41 known miRNAs and 18 novel miRNAs were identified in the exosome-like vesicles. Eleven selected miRNAs, including 7 known miRNAs (miR-71-5p, miR-10a-5p, miR-let-7-5p, miR-745-3p, miR-219-5p, miR-124-3p and miR-4989-3p) and 4 novel miRNAs (novel-mir-3, novel-mir-7, novel-mir-8 and novel-mir-11) were validated to exist in metacestiodes and exosome-like vesicles of *T. pisiformis* cysticercus by qPCR. The functions of most targets of exosomal miRNAs were mainly associated with signal transduction and the immune system. Additionally, *T. pisiformis* cysticercus-derived vesicles induced the production of IL-4, IL-6, IL-10, IL-13 and Arg-1, but downregulated the expression of IL-12, IFN-γ and iNOS in RAW264.7 macrophages.

**Conclusions:**

We demonstrated that proteins and miRNAs enclosed within exosome-like vesicles from *T. pisiformis* cysticercus have immunomodulatory functions. Furthermore, exosome-like vesicles were shown to induce the macrophage Th2-type immune response *in vitro*. Our study suggests that exosome-like vesicles play an important role in the interaction between cysticerci and their hosts.
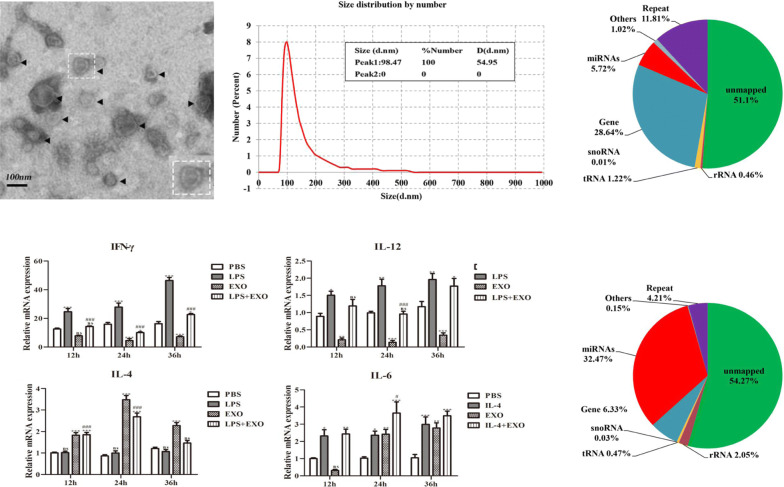

## Background

*Taenia pisiformis*, a common intestinal tapeworm in canines and felines, is widely distributed around the world [1, 2]. Cysticercus pisiformis, the larval stage of *T. pisiformis*, causes considerable economic losses to the rabbit breeding industry [3]. Infection in the definitive host may occur when they consume lagomorph internal organs infected with *T. pisiformis* cysticercus. *Oryctolagus cuniculus* become infected through ingestion of water or forage contaminated with *T. pisiformis* eggs. Cysticerci usually parasitize the liver capsule, peritoneum, greater omentum and mesentery, and occasionally other organs such as the pelvis or lungs [3, 4]. Rabbits infected with *T. pisiformis* have a weakened immunologic resistance and are susceptible to secondary infections with other pathogens, which may cause host digestive disorders, growth retardation, a decrease in feed conversion, reduction of proliferation, and even death [5].

Helminth infections are generally characterized by a polarized type-2 immune response, which could be initiated by helminth excretory/secretory products (ESP). Recently, helminth-derived exosomes or extracellular vesicles (EVs) have been proven to be a new paradigm in the study of parasite-host communication [6, 7]. Exosomes are nano-scale (30–150 nm) membrane-bound vesicles that are released into the extracellular environment *via* the fusion of the plasma membrane with the multi-vesicular bodies (MVBs) [8–10]. These vesicles are produced by normal [11] and pathological cells [12] and can be obtained from extracellular fluids, including urine [13], blood plasma [14], breast milk [15], saliva [16], and cerebrospinal fluid [17]. Exosomes carry a diverse suite of bioactive molecules, including nucleic acids, proteins and lipids, which can be transferred into target cells to mediate intercellular communication [18–21]. Exosome-like vesicles from helminths may play a pivotal role in parasitic infection [22–25]. Recent studies of several cestodes have revealed that exosome-like vesicles from parasites can deliver miRNAs or proteins cargo into host cells to modulate the host immune system [10, 26]. *Echinococcus granulosus* cyst fluids contain some parasite-derived EVs and specific proteins, some of which were associated with cyst survival [27]. *Echinococcus multilocularis* was shown to release exosome-derived miR-71 into the host and affected the function of macrophages [28]. Moreover, *Heligmosomoides polygyrus*-derived EVs have been shown to be taken up by murine macrophages and inhibited their activation [29]. These studies suggest that exosome-like vesicles take part in parasite-host interactions through delivering specific functional components. However, the information about the contents of cysticercus pisiformis-derived exosome-like vesicles and their functional effects on macrophages remains largely unknown. Therefore, the purpose of the present investigation was to profile the proteins and miRNA components of cysticercus pisiformis-derived exosome-like vesicles and to explore the role of exosomes-like vesicles in macrophages in metacestode infection.

## Methods

### Animals and parasites

Animals used in this study were purchased from the Laboratory Animal Center of Lanzhou Veterinary Research Institute. New Zealand white rabbits were housed individually in wire cages equipped with a plastic nest and had access to food and water *ad libitum.* Fresh *T. pisiformis* cysticerci were harvested from New Zealand white rabbits 50 days after infection with 500 eggs of *T. pisiformis*.

### Exosome-like vesicles isolation

To prepare exosome-like vesicles derived from *T. pisiformis* cysticercus, rabbits infected with *T. pisiformis* cysticerci were sedated with xylazine (5 mg/kg) and ketamine (25 mg/kg), and euthanized with a lethal dose of sodium pentobarbital (100 mg/kg). Metacestodes collected from the peritoneum and greater omentum of rabbits were washed thoroughly in sterile 0.9% sodium chloride containing 100 μg/ml streptomycin and 100 IU/ml penicillin (Life Technologies, Grand Island, NY, USA). The larvae were washed three times with RPMI-1640 culture medium (Invitrogen, Carlsbad, CA, USA) and maintained in T25 flasks in RPMI-1640 medium supplemented with 10% exosome-depleted fetal bovine serum (FBS), 100 μg/ml streptomycin and 100 IU/ml penicillin at 37 °C under 5% CO_2_. Each flask contained 50 cysts in 15 ml culture medium. To ensure host components were expelled thoroughly from larvae, the medium was changed after 12 h [30]. ESP from *T. pisiformis* cysticerci were obtained at 24 h and 48 h and stored at 4 °C prior to centrifugation.

Exosome-like vesicles from the ESP of *T. pisiformis* cysticerci were purified by serial centrifugation as previously described [31]. 100 ml pooled ESP from cysticercus pisiformis were subjected to two successive centrifugations at 300× *g* for 10 min and 10,000× *g* for 30 min to remove cellular debris and dead cells. The supernatant was harvested and centrifuged at 75,000× *g* at 4 °C for 90 min to remove large vesicles. The supernatant was collected and centrifuged at 110,000× *g* for 90 min at 4 °C. The resultant pellet was re-suspended in 12 ml filtered PBS, then centrifuged at 110,000× *g* for 90 min to remove remaining protein contaminants, and re-suspended in 50 μl PBS. The concentration of purified exosomal proteins was determined using a Pierce BCA Protein Assay Kit (Thermo Fisher Scientific, Waltham, MA, USA). All aliquots were stored at -80 °C until further use.

### Transmission electron microscopy (TEM)

The morphology and size of exosome-like vesicles from cysticercus pisiformis were visualized by TEM. Briefly, 10 μl of exosomes from cysticercus pisiformis were loaded onto a 200-mesh formvar-coated copper grid (Agar Scientific Ltd, Stansted, UK) for 10 min and the excess stain was removed by blotting with filter paper. Exosome-like vesicle pellets were negatively stained with a 3% solution of phosphotungstic acid (pH 7.0) for 1 min at room temperature. Grids were air-dried and imaged using a Hitachi TEM at a voltage of 80 kV.

### Nanoparticle tracking analysis (NTA)

The size distribution and number of exosome-like vesicles were analyzed by measuring the rate of Brownian motion of each particle using a NanoSight LM10 instrument (Nanosight, Wiltshire, UK). The LM10 uses digital cameras to directly track the movement of individual particles in solution, thereby enabling the determination of particle size distribution as well as the number of nanoparticles [32]. The measurement procedure was performed as previously described [33]. Each sample was measured in triplicate and the NTA analytical software (version 2.3; Nanosight, Wiltshire, UK) was used to capture and analyze the data.

### Mass spectrometry analysis

To identify the proteins of exosome-like vesicles from *T. pisiformis* cysticercus, three biological replicates were prepared as described above. Each 10 μg pelleted exosome was lysed with 150 μl RIPA lysis buffer (Beyotime, Shanghai, China) and separated by 12% polyacrylamide gel electrophoresis (PAGE), respectively. All bands were cut into 1 mm^3^ cubes and washed thrice with 25 mM NH_4_CO_3_ in 50% acetonitrile (ACN) for 1 h, and subjected to dehydration with ACN and reduction with 10 mM dithiothreitol (DTT) at 56 °C for 1 h. Alkylation was carried by the addition of 55 mM iodoacetamide (IAM) at room temperature for 45 min. In-gel digestion was performed using 2.5 μg trypsin at a ratio (w/w) of 1:40 (enzyme:substrate) at 37 °C overnight and was stopped with 5% formic acid (FA). Peptides were desalted with a Waters Oasis HLB column (Waters, Milford, MA, USA) and eluted in 2% ACN and 0.1% FA before drying with a Benchtop Centrifugal Vacuum Concentrator (Labconco, Kansas City, MO, USA). Peptides were subjected to LC-20AD nano-HPLC (Shimadzu, Kyoto, Japan) spectrometry for peptide separation and data analysis. Briefly, samples were loaded onto a C18 trap column at 15 μl/min in solvent A (2% ACN, 0.1% FA) and the peptides were eluted and loaded onto an analytical column using a 44 min gradient, from 2% to 35% solvent B (98% ACN, 0.1% FA), at a flow rate of 400 nl/min. The eluate was subjected to nanoelectrospray ionization followed by tandem mass spectrometry in a Q-Exactive Orbitrap mass spectrometer (Thermo Fisher Scientific). Relative parameters were set as a positive ion mode and data dependent mode with full MS scans from 200 to 1800 m/z, resolution at 70,000, normalized collision energy at 27, charge state at 2 +, 3 +, 4 + and 5 +, and resolution at 17,500. After the survey scans, the top 15 most abundant precursor ions were fragmented by high-energy collision dissociation (HCD). Since genome information on *T. pisiformis* was not yet obtained, the MS data analysis was carried out by Mascot software (version 2.3.02; Matrix Science, London, UK) using genomes from three parasites that have high kinship with *T. pisiformis*, including *T. solium* (http://www.genedb.org/Homepage/T.solium), *E. granulosus* (ftp://ftp.ncbi.nlm.nih.gov/genomes/all/GCA/000/524/195/GCA_000524195.1_ASM52419v1/) and *E. multilocularis* (ftp://ftp.ncbi.nlm.nih.gov/genomes/all/GCA/000/469/725/GCA_000469725.3_EMULTI002/). Additionally, common Repository of Adventitious Proteins (cRAP) (http://www.thegpm.org/crap/) was used to analyze proteins commonly found in proteomic experiments and *Oryctolagus cuniculus* genome database (https://www.uniprot.org/uniprot/?query=taxonomy:9986) was used to remove the protein from hosts. Database search parameters were set as follows: trypsin as enzyme; peptide mass tolerance of 20 ppm and fragment mass tolerance of 0.05 Da; + 1, + 2, + 3 as peptide charge; a maximum of one missed cleavage; carbamidomethyl (C), iTRAQ8plex (N-term), iTRAQ8plex (K) as fixed modifications and oxidation (M), Gln- > pyro-Glu (N-term Q), deamidated (NQ) as variable modifications. False discovery rate (FDR) lower than 0.01 was used as a screening condition [34, 35].

Gene ontology (GO) analysis of the identified proteins was conducted using the Gene ontology database (http://www.geneontology.org). Functional annotations of the proteins were performed using the Blast2GO program (https://www.blast2go.com) against the non-redundant protein database (NCBInr). Additionally, the Clusters of Orthologous Groups (COG) database (http://www.ncbi.nlm.nih.gov/COG/) and Kyoto Encyclopedia of Genes and Genomes (KEGG) database (http://www.genome.jp/kegg/) were used to classify and group these identified proteins.

### Western blot analysis

The protein concentration of exosome-like vesicles, soluble worm antigens (SAg) and ESP from *T. pisiformis* cysticercus were measured using a BCA protein assay kit. Fifteen μg of total protein was denatured at 100 °C for 10 min and separated by 12% SDS-PAGE. The proteins were transferred to polyvinylidene fluoride membranes (Millipore, Burlington, MA, USA) for 13 min and blocked with 5% non-fat milk in PBST for 2 h at room temperature. Two antibodies of anti-14-3-3 and anti-enolase (1:200; both from *T. solium* produced in rabbits and were prepared in our laboratory) [36] were separately added to the membrane and incubated at 4 °C overnight. The membranes were washed three times with PBST and incubated with HRP-goat-anti rabbit IgG (H + L) (1:1000; Beyotime, Shanghai, China). The bands were developed using an ECL chemiluminescence working solution (Beyotime) following the manufacturer’s instructions.

### RNA extraction and high-throughput small RNA sequencing

Exosome-like vesicles derived from fresh *T. pisiformis* cysticerci (served as a positive control) in three biological replicates were prepared as described above, and total RNA was extracted using TRIzol reagent (Invitrogen). RNA sample integrity and quality were determined using an Agilent 2100 Bioanalyzer (Agilent Technologies, Santa Clara, CA, USA). High-throughput small RNA sequencing was carried out by BGI (Shenzhen, China). Briefly, RNA fragments (18–30 nt) were separated by PAGE. After ligation of 3’ and 5’ adaptors to both ends of small RNAs, the ligation products were used for reverse transcription PCR. The PCR products (100–120 bp) were further separated on a PAGE gel and small RNA sequencing libraries were generated using a TruSeq Small RNA Library Preparation Kit (Illumina, San Diego, CA, USA) following the manufacturer’s protocol. Library sequencing was carried out on an Illumina HiSeq 2500 system (Illumina), and small RNA clean reads were obtained after removing adaptor reads, low quality reads and contaminants. Because of the unavailability of *T. pisiformis* genome, we used the *T. solium* genome (https://parasite.wormbase.org/Taenia_solium_prjna170813/Info/Index/), *E. granulosus* (ftp://ftp.ncbi.nlm.nih.gov/genomes/all/GCA/000/524/195/GCA_000524195.1_ASM52419v1/) and *E. multilocularis* (ftp://ftp.ncbi.nlm.nih.gov/genomes/all/GCA/000/469/725/GCA_000469725.3_EMULTI002/) as references to align screened small RNA sequences (18–30 nt). Afterwards, the mapped reads were aligned to the miRBase database (http://mirbase.org) and *Echinococcus* spp. metacestode miRNA dataset to annotate known miRNAs (E-value < 0.05). Small RNA expression profiles, including miRNA, snRNA, snoRNA, tRNA, rRNA, and piRNA, were annotated using the RFam database (http://rfam.janelia.org). The RepBase database (http://www.girinst.org/repbase) and the pre-setting reference genome database were also used to identify small RNAs. In addition, the unannotated sequences were used to predict potential novel miRNA candidates through searching the characteristic hairpin structure of the miRNA precursor [37]. The prediction of targets of exosomal miRNAs was conducted using RNAhybrid, miRanda and TargetScan software. The potential biological functions of target genes were predicted using the KEGG database.

### Macrophage cell culture and treatment

RAW264.7 murine macrophage cell lines were maintained in Dulbecco’s modified Eagle’s medium (DMEM) supplemented with 10% FBS and cultured in a 37 °C incubator with 5% CO_2_. The cells were plated in 6-well plates (1 × 10^6^ cells/ml) and incubated with exosome-depleted DMEM conditioned media containing either 200 ng/μl LPS (Sigma-Aldrich, St. Louis, MO, USA), 40 ng/ml IL-4 (Sigma-Aldrich), 50 μg/ml *T. pisiformis* cysticercus-derived exosome-like vesicles, sterile PBS, or a combination with LPS + exosome-like vesicles, or IL-4 + exosome-like vesicles. All treatments were conducted in triplicate.

### Quantitative real-time PCR (qPCR) for miRNAs and mRNAs

miRNAs from 50 μl exosome-like vesicles of *T. pisiformis* cysticercus and 20 mg *T. pisiformis* cysticerci tissue were extracted using an miRNeasy kit (Qiagen, Germantown, MD, USA) following the manufacturer’s instructions. The first-strand cDNA was synthesized using 2 μg of total miRNA using Mir-X™ miRNA First-Strand Synthesis Kit (Takara, Shiga, Japan) according to the manufacturer’s protocols. The qPCR reaction system consisted of 12.5 μl of 2× TB Green Advantage Premix, 0.5 μl of 50× ROX Dye, 0.5 μl of miRNA-specific forward primer, 0.5 μl of universal miRNA reverse primer, 2 μl of cDNA and 9 μl of ddH_2_O. qPCR reactions were performed on an ABI7500 instrument (Thermo Fisher Scientific, Waltham, MA, USA) according to the following parameters: initial activation at 95 °C for 30 s followed by 40 cycles of 95 °C for 5 s and 60 °C for 34 s. Data was evaluated using online software (http://pcrdataanalysis.sabiosciences.com/mirna). All miRNA primers were purchased from Guangzhou RiboBio Co., Ltd (Guangzhou, China) (Additional file 1: Table S1). As a reference, cel-miR-39-3p was added to each sample to monitor miRNA extraction efficiency and normalize sample-to-sample variation. The relative abundance of miRNAs was calculated and normalized using the 2^−ΔΔCq^ method.

To determine the relative expression of cytokines in RAW264.7 macrophages during *T. pisiformis* cysticercus exosome-like vesicles stimulation, total RNA from differently treated cells were isolated and probed by qPCR using mouse-specific primers (IL-4, IL-6, IL-10, IL-12, IL-13, Arg-1, iNOS, and the housekeeping gene GAPDH) (Genecopoeia, Guangzhou, China) (Additional file 1: Table S2). The qPCR was conducted using the TransScript Green One-Step qPCR SuperMix (TransGen Biotech, Beijjing, China) on an ABI7500 instrument. The qPCR reaction system consisted of 10 μl of 2× TransStart Tip Green qPCR SuperMix, 0.4 μl of TransScript One-Step RT Enzyme Mix, 0.4 μl of Passive Reference Dye, 0.8 μl of forward primer, 0.8 μl of reverse primer, 2 μl of RNA template and 5.6 μl of ddH_2_O. qPCR reaction procedures and data analysis were performed as previously described.

### Enzyme-linked immunosorbent assay (ELISA)

Following RAW264.7 cell stimulation with LPS, IL-4, exosome-like vesicles from *T. pisiformis* cysticercus, PBS, or a combination for 12 h, 24 h and 36 h, the cell-free supernatants were harvested and frozen at -80 °C until the assay was performed. The levels of Th1 and Th2 cytokines in the supernatant was assessed using commercially available mouse cytokine (IL-4, IL-6, IL-10, IL-13, IL-12 and IFN-γ) ELISA kits (RayBiotech, Peachtree Corners, GA, USA) according to the manufacturers’ protocols. Each experiment was performed in triplicate.

### Statistical analyses

Statistical analyses were conducted using GraphPad Prism 5.0. Comparisons between groups were assessed using the unpaired Student’s t-test. Differences among multiple groups were analyzed by a one-way analysis of variance (ANOVA) using SPSS 24.0 (SPSS Inc., Chicago, IL, USA). Data are presented as the mean ± standard error of the mean (SE). Statistical significance is indicated as *P < 0.05, **P < 0.01 and ***P < 0.001.

## Results

### Size and morphological analysis of *T. pisiformis* cysticercus*-*derived exosome-like vesicles

**Fig. 1:**
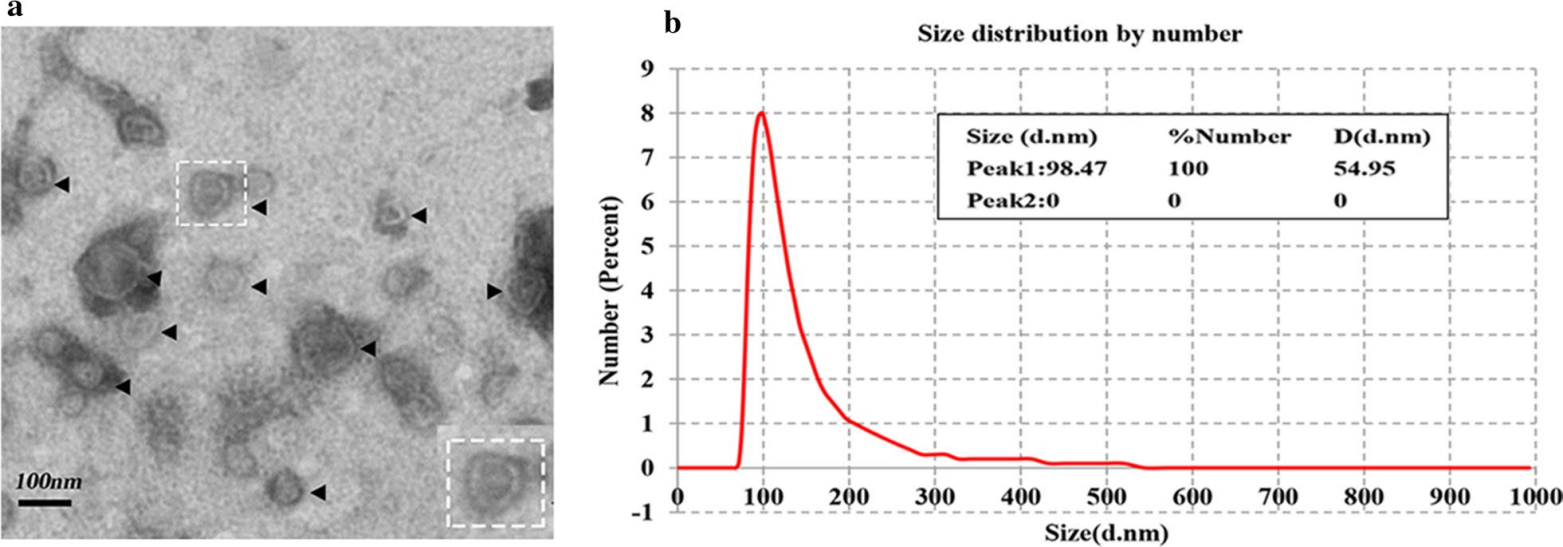
Characterization of exosome-like vesicles secreted by *T. pisiformis* cysticercus. **a** Morphological characterization of *T. pisiformis* cysticercus exosome-like vesicles by TEM. The arrowheads indicate exosome-like vesicles stained with phosphotungstic acid. The dotted box portion represents a vesicle with a bi-layered membrane. **b** NTA diameter distribution analysis of the purified exosome-like vesicles

To confirm the presence of exosome-like vesicles isolated from the culture medium of *T. pisiformis* cysticercus, the pellets obtained from sequential centrifugation were subjected to TEM and NTA analysis. TEM images showed that these vesicles were spherical, approximately 30–150 nm in diameter and with lipid bilayer-bound membrane structures (Fig. 1a). The particle size distribution of the vesicles was distributed around 50–150 nm and peaked at a mean diameter of 98.47 nm (Fig. 1b), which had the prototypical size characteristic of exosome-like vesicles and was consistent with exosomes from other parasites [10, 23, 27, 38–40]. This data indicated that we successfully isolated and purified exosome-like vesicles from *T. pisiformis* cysticercus.

### Characterization of *T. pisiformis* cysticercus exosomal protein cargo

**Fig. 2:**
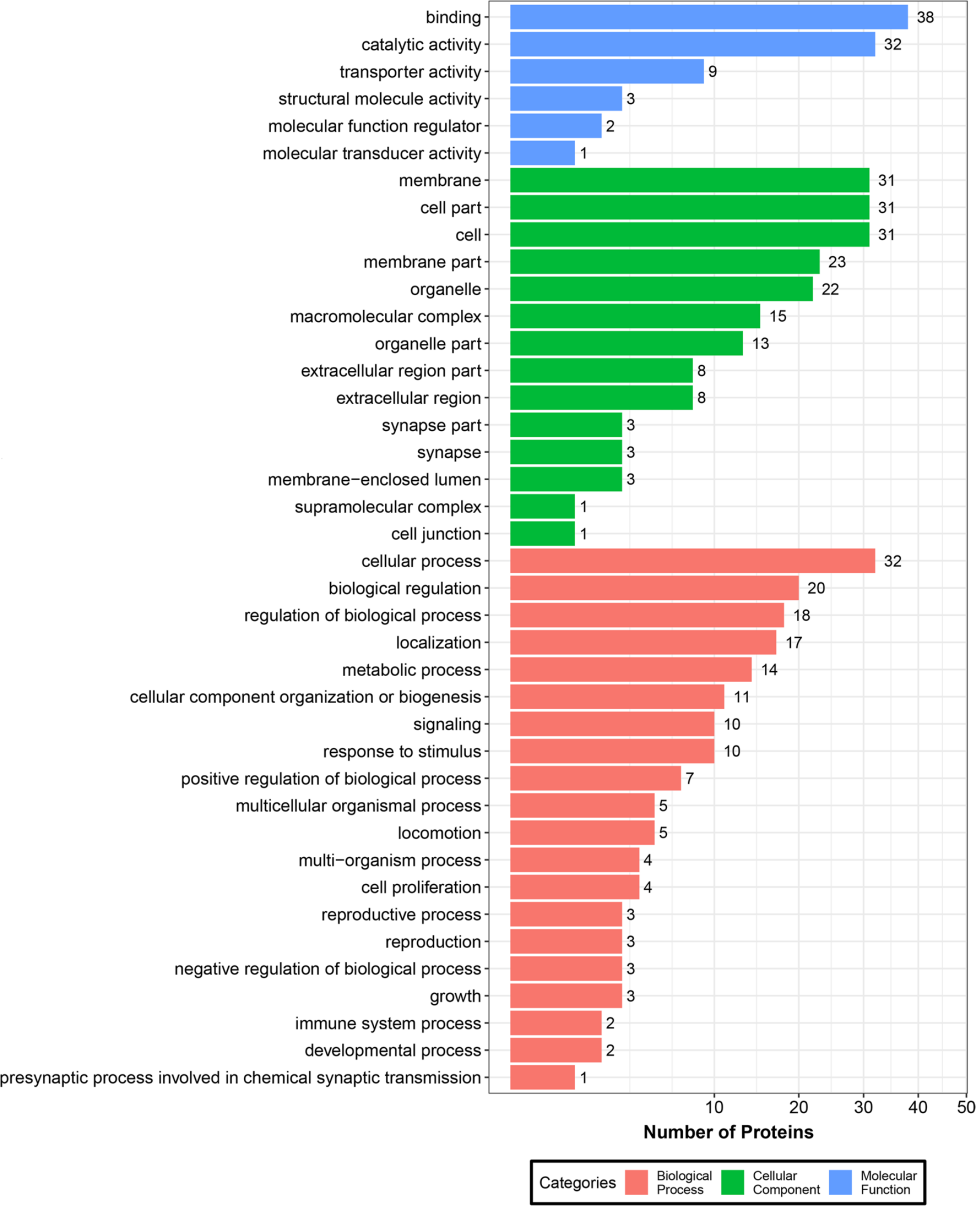
Functional enrichment analysis of protein cargo enriched in *T. pisiformis* cysticercus exosome-like vesicles. Bar plot showing biological process, cellular component, and molecular function GO categories in exosome-like vesicles derived from *T. pisiformis* cysticercus

The MS/MS analysis identified 87 parasite-unique proteins in *T. pisiformis* cysticercus-derived exosome-like vesicles (Additional file 2: Table S3). GO analysis showed that these proteins were classified into 40 categories by cellular component, biological process, and molecular function (Fig. 2, Additional file 2: Table S4). In terms of the cellular component, the proteins were mostly related to the membrane (16.06%), cell part (16.06%) and membrane part (11.92%). Biological process studies suggested that these exosomal proteins were involved in the cellular process (18.39%), biological regulation (11.49%) and regulation of biological processes (10.34%). In addition, most of molecular functions were classified into three categories: binding (40.43%), catalytic activity (34.04%) and transporter activity (9.57%) in the identified proteins. The top 50 parasite-origin proteins with unique spectra numbers ≥ 3 are presented in Table 1, and some of these were identified as the most common exosomal proteins in Exocarta, mainly including chaperones (heat-shock protein and beta-soluble NSF attachment protein), cytoskeletal proteins (actin, Rab and tubulin), metabolic enzymes (enolase, phosphoenolpyruvate carboxykinase and fructose 1, 6 bisphosphate aldolase), molecules associated with signal transduction (annexin, 14-3-3 proteins, programmed cell death 6 interacting protein), elongation factor 1-alpha, and phosphoglycerate kinase. Most of these parasite-origin proteins have been described in exosome-like vesicles from *Echinococcus* and other flatworm parasites [27, 41, 42].

**Table 1:** List of top 50 parasite-derived proteins identified in the proteome of exosome-like vesicles from *T. pisiformis* cysticercus

Accession	Annotation	No. of unique peptides	No. of unique spectra
Chaperones			
EUB56318.1	**Heat shock cognate protein**	6	9
EUB64499.1	Beta-soluble NSF attachment protein	4	4
Cytoskeletal/Structural proteins			
EUB56079.1	**Actin-1**	9	10
EUB64035.1	Moesin/ezrin/radixin	7	10
EUB54636.1	Rab	6	9
EUB61143.1	Tubulin beta-3 chain	6	6
EUB57627.1	Rab GDP dissociation inhibitor alpha	4	4
Extracellular matrix/secrected proteins			
EUB61528.1	Basement membrane-specific heparan sulfate proteoglycan core protein	5	5
EUB57979.1	Exocyst complex component 3	5	5
Hypothetical proteins			
EUB54981.1	Putative phospholipid-transporting ATPase IIB	13	14
EUB54777.1	Hypothetical protein EGR_10354	6	6
Metabolic Enzymes			
CDS19796.1	**Enolase**	9	9
CDS18138.1	V type proton ATPase 116 kDa subunit a	5	6
CDS15195.1	Tryptophanyl tRNA synthetase	4	5
EUB61757.1	Peptidyl-prolyl cis-trans isomerase	4	5
tr|W8P1J2|W8P1J2_TAESO	Phosphoenolpyruvate carboxykinase	4	6
tr|D2U5C3|D2U5C3_TAESO	Long chain fatty acid coenzyme A ligase 5	3	4
EUB60416.1	Long-chain-fatty-acid–CoA ligase	3	4
CDS17202.1	Fructose 1 6 bisphosphate aldolase	3	3
Protease			
EUB64462.1	Calpain-A	10	10
CDS21212.1	cGMP dependent protein kinase	9	10
CDS24569.1	Intestinal type alkaline phosphatase 1	4	4
EUB59398.1	**Phosphoglycerate kinase**	3	4
EUB58966.1	Ras gtpase	3	4
Signal Transduction and Biological Regulation			
EUB64724.1	**Annexin**	6	6
EUB62107.1	**14-3-3 protein**	5	5
EUB64038.1	Vacuolar protein sorting-associated protein 4A	6	6
EUB59108.1	Transforming protein RhoA	4	6
EUB63467.1	Receptor Mediated Endocytosis family member	5	5
CDS21096.1	Endophilin B1	4	5
EUB57999.1	Ras-related C3 botulinum toxin substrate 2	4	5
EUB64797.1	Ras-related protein O-RAL	4	5
EUB63534.1	Ras-related protein Rap-1b	4	4
EUB64384.1	**Programmed cell death 6 interacting protein**	4	4
EUB59848.1	ADP-ribosylation factor	4	4
EUB60984.1	**Annexin A6**	3	4
Transporters/Channels			
EUB57119.1	Sodium/potassium-transporting ATPase subunit alpha	20	20
CDS23982.1	Major vault protein	9	9
EUB60605.1	Solute carrier family 5	5	6
CDS22870.1	Lipid transport protein N terminal	5	6
EUB61207.1	BRO1 domain containing protein BROX	4	5
EUB62853.1	Ras-related protein Rab-2A	4	4
EUB62794.1	Otoferlin	4	4
Others			
EUB53928.1	Ubiquitin	6	13
EUB62510.1	**Elongation factor 1-alpha**	7	8
EUB62111.1	Tetracycline resistance leader peptide TetL	6	6
EUB64561.1	Multidrug resistance protein	6	6
EUB63692.1	Myoferlin	5	6
EUB59451.1	NEDD4 E3 ubiquitin protein ligase WWP1	4	6
EUB59351.1	Tegumental proteins	3	3

*Note*: Both unique peptide number and unique spectra number ≥ 3 are listed. Proteins listed in bold font represent the most common proteins of the “top 30” exosomes-like vesicles in ExoCarta

### Validation of *C. pisiformis* exosome-like vesicles proteomics results

**Fig. 3:**
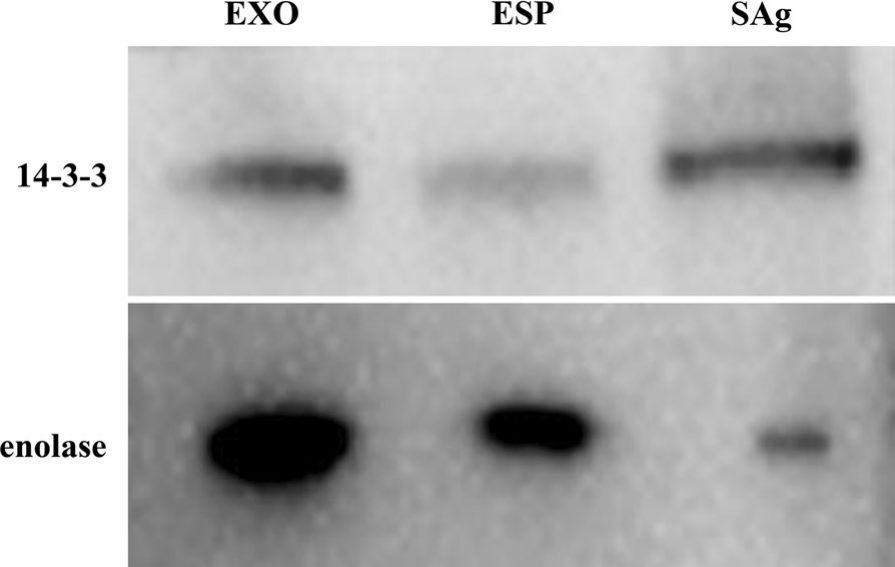
Western blot of exosomal markers 14-3-3 and enolase. Western blot revealed 14-3-3 with an expected size of approximately 28 kDa and enolase with an expected size of approximately 54 kDa. Both ESP and SAg served as positive controls

To verify the proteomic data, two exosome-like vesicle enriched proteins (14-3-3 and enolase) were used for confirmation by western blotting. The results showed that these proteins were detected in the exosome-like vesicles, ESP and SAg of *T. pisiformis* cysticercus (Fig. 3), consistent with the MS result that 14-3-3 and enolase were enriched in exosome-like vesicles from *T. pisiformis* cysticercus.

### Small RNA sequencing data analysis and qPCR validation

**Fig. 4:**
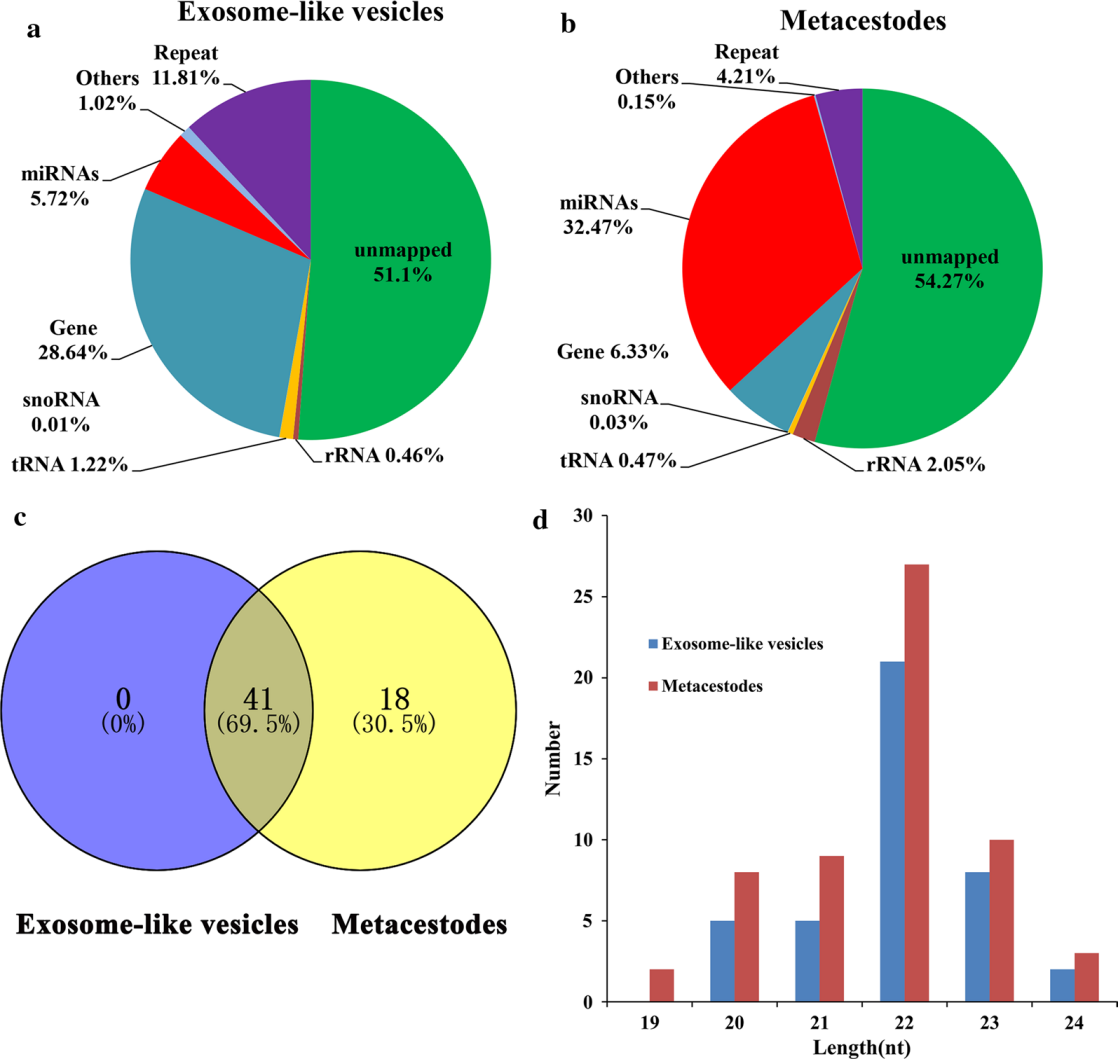
Length distribution of annotated miRNAs in exosome-like vesicles and cysticercus of *T. pisiformis*. **a**, **b** All the mapped clean reads were annotated, including miRNAs, rRNAs, tRNAs, snRNAs and snoRNAs. Pie-charts of annotated small RNAs and their percentages in exosome-like vesicles and cysticercus of *T. pisiformis*. **c** Common miRNAs in exosome-like vesicles and cysticercus of *T. pisiformis*. **d** Length distribution of identified miRNAs

To identify the small RNA components in exosome-like vesicles from *T. pisiformis* cysticercus, total RNA extracted from exosome-like vesicles and larvae of *T. pisiformis* were analyzed using small RNA high-throughput sequencing. The results revealed that a total of 36,961,810 and 31,592,685 raw reads were identified in exosome-like vesicles and metacestodes from the small RNA sequencing library, respectively. After filtering and processing, a total of 24,329,002 (65.82%) and 24,897,949 (77.81%) clean reads were obtained. Among these reads, approximately 14,568,998 (59.88%) and 6,804,366 (27.66%) reads were mapped to the reference genome database. All mapped reads were used for small RNA classification, including miRNA, tRNA, rRNA, snRNA and snoRNA (Fig. 4a, b).

A total of 41 and 59 known miRNAs were identified in two libraries. Among them, all of the miRNAs identified in exosome-like vesicles were found in larvae libraries (Fig. 4c, Additional file 3: Table S5, Table S6). The miRNA length distribution of the two libraries showed that almost all of miRNAs were 20–24 nt (Fig. 4d) and the predominant species was 22 nt, a typical length of Dicer-processed products, which was consistent with the previous reports in other cestodes [30, 43–45]. Among identified exosomal miRNAs, the most abundant miRNAs were miR-277, followed by miR-10 and miR-71 (Additional file 3: Table S5). Furthermore, 18 novel miRNAs were successfully predicted in the exosome-like vesicles and metacestodes using Mireap (Additional file 3: Table S7).

**Fig. 5:**
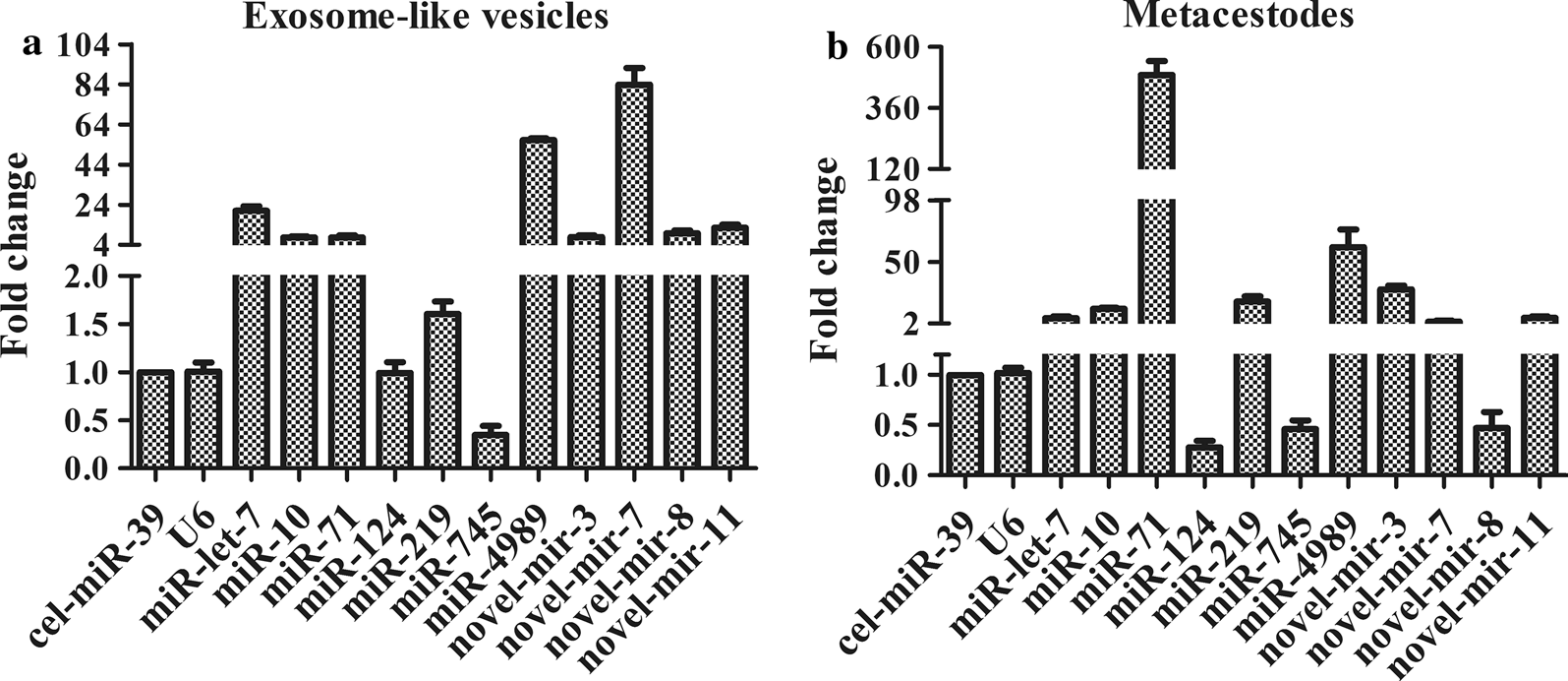
qPCR identification of relevant expression of miRNAs in exosome-like vesicles and cysticercus of *T. pisiformis*. **a** Relevant expression of miRNAs in *T. pisiformis* cysticercus exosome-like vesicles. **b** Relevant expression of miRNAs in *T. pisiformis* cysticercus. Cel-miR-39-3p served as an external control to normalize sample-to-sample variation

To validate exosomal small RNA sequencing data, eleven miRNAs with differing abundance were selected for qPCR analysis. Of these miRNAs, the average read counts of miR-10a-5p, miR-219-5p and miR-124b-3p were 28,517, 995 and 130, respectively, which represented the high, middle and low abundance levels of the identified miRNAs (Additional file 4: Table S5). The quantification results showed that the relative expression levels of 7 known miRNAs (miR-71-5p, miR-10a-5p, miR-let-7-5p, miR-745-3p, miR-219-5p, miR-124-3p and miR-4989-3p) and 4 novel miRNAs (mir-3, mir-7, mir-8 and mir-11) were consistent with those in RNA sequencing (Fig. 5), indicating the accuracy and reliability of the exosomal miRNAs sequencing data.

### Bioinformatics analysis of known miRNAs in exosome-like vesicles

**Fig. 6:**
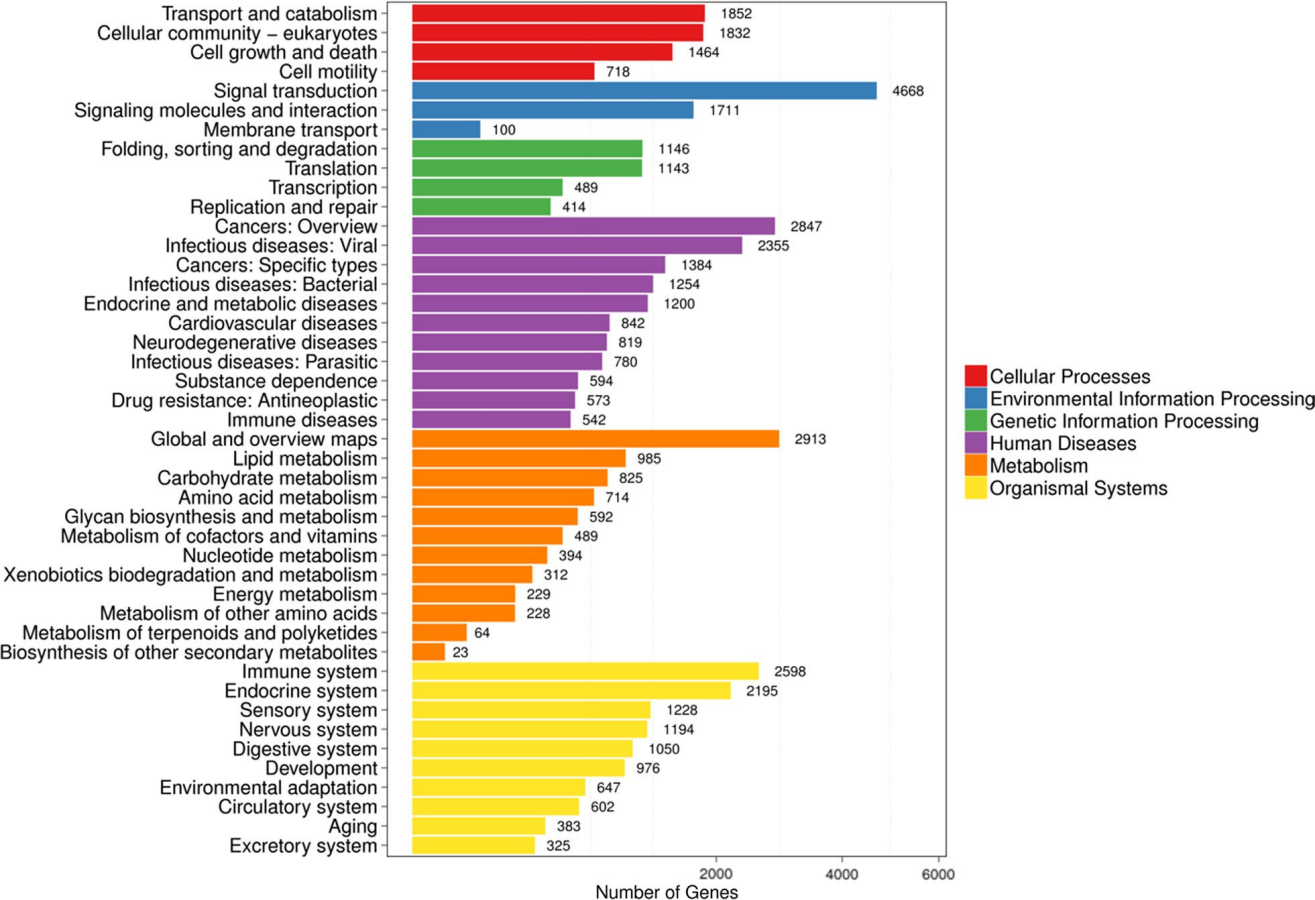
KEGG pathway classification and functional enrichment of predicted target genes of exosomal miRNAs. The number of proteins in each category is indicated next to the bars

To determine the potential biological functions of the targets of the exosome-like vesicle miRNAs of cysticercus pisiformis, the targets of the exosomal miRNAs were predicted using RNAhybrid and miRanda software packages. A total of 99,278 targets of 41 miRNAs were identified (Additional file 4: Table S8). KEGG analysis revealed that the potential biological functions of most targets were involved mainly in signal transduction and immune system, except for cancer, global and overview maps (Fig. 6). Moreover, some of well-known immune-related miRNAs were identified in exosome-like vesicles, including miR-2a, miR-9, miR-10a, miR-71 and let-7-5p [23, 28, 46–48]. Therefore, we speculated that the *T. pisiformis* cysticercus-derived exosome-like vesicles might be involved in modulating host immune response by delivering immune-related miRNA content.

### *Taenia pisiformis* cysticercus exosome-like vesicles stimulated secretion of molecules related to the Th2-type immune response in macrophages

**Fig. 7:**
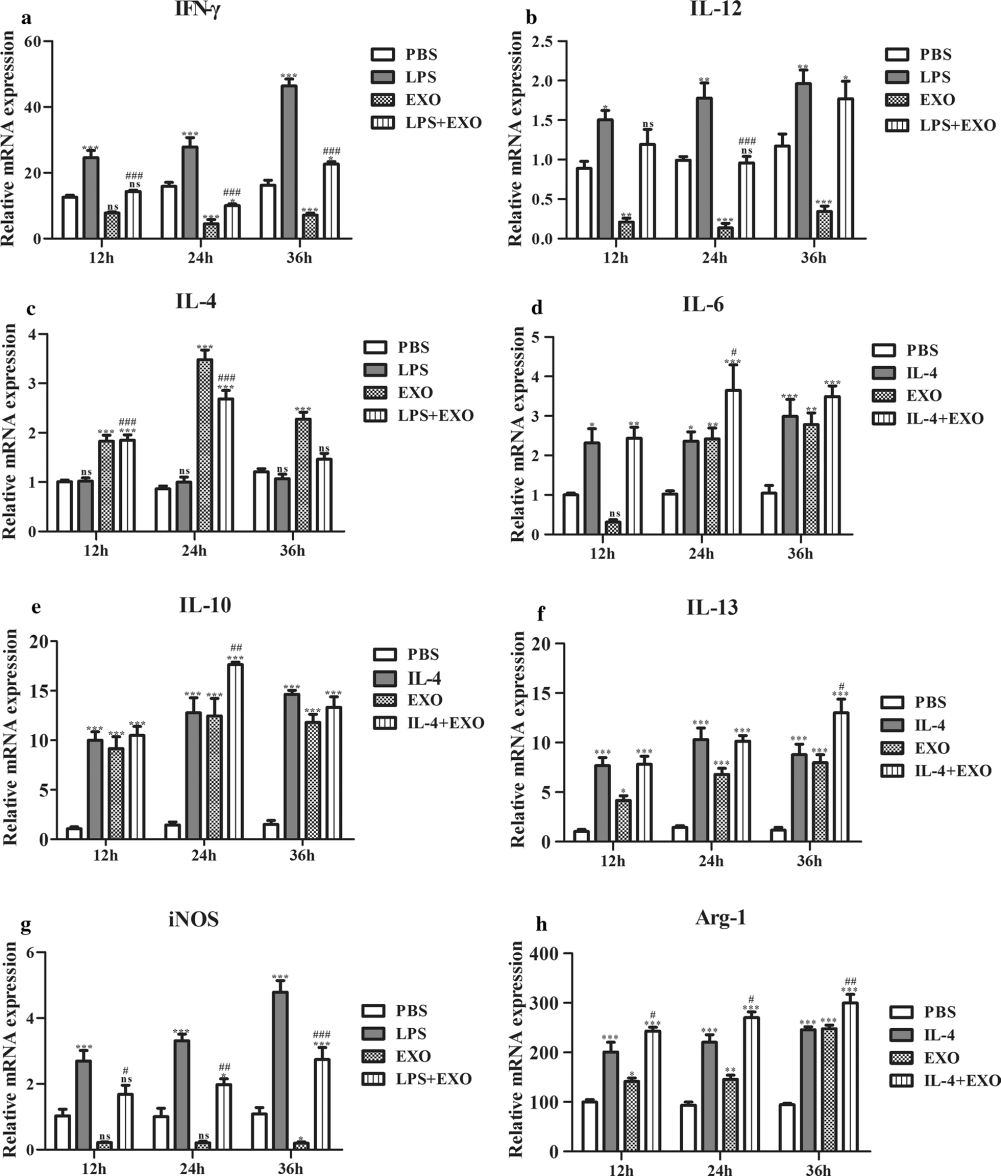
Fold regulation (qPCR) in M1 and M2 cytokine gene expression induced by *T. pisiformis* cysticercus exosome-like vesicles in RAW264.7 macrophages. LPS and IL-4 served as positive controls for M1 phenotype and M2 phenotype molecules, respectively. PBS served as the negative control for cytokine stimulaton. **a**, **b**, **g** Induction of M1 markers in RAW264.7 macrophages treated with *T. pisiformis* cysticercus exosome-like vesicles. **c**-**f**, **h** Induction of M2 markers in RAW264.7 macrophages by *T. pisiformis* cysticercus exosome-like vesicles. Data for the final analysis are from three independent experiments and are expressed as the mean ± standard error (SE). *P < 0.05, **P < 0.01 and ***P < 0.001 were considered statistically significant compared to PBS-treated RAW264.7 macrophages. ^#^*P* <  0.05, ^##^*P* < 0.01 and ^###^*P* < 0.001 were considered statistically significant compared to LPS/IL-4 treated RAW264.7 macrophages (Additional file 5: Table S9)

To investigate the potential function of exosome-like vesicles as determined by bioinformatics, we examined the *in vitro* effects of exosome-like vesicles on the release of Th1- and Th2-associated bioactive molecules in RAW264.7 macrophages. The qPCR results showed that the mRNA levels of IFN-γ and iNOS at 12 h, 24 h and 36 h increased significantly in LPS-activated macrophages (Fig. 7a, b, g). The expression of IL-6, IL-10, IL-13 and Arg-1 increased significantly in IL-4-activated macrophages (Fig. 7d–f, h). The mRNA levels of IFN-γ and iNOS at 12 h, 24 h and 36 h and IL-12 at 24 h decreased significantly in the LPS + EXO group compared to the LPS group (Fig. 7a, b, g), while expression of Arg-1 at 12 h, 24 h and 36 h, IL-4 at 12 h and 24 h, IL-6 at 24 h, IL-10 at 24 h and IL-13 at 36 h increased significantly in the LPS + EXO group compared with the LPS group (Fig. 7c–f). These data showed that *T. pisiformis* cysticercus exosome-like vesicles primed macrophages to secrete Th2 related bioactive molecules.

**Fig. 8:**
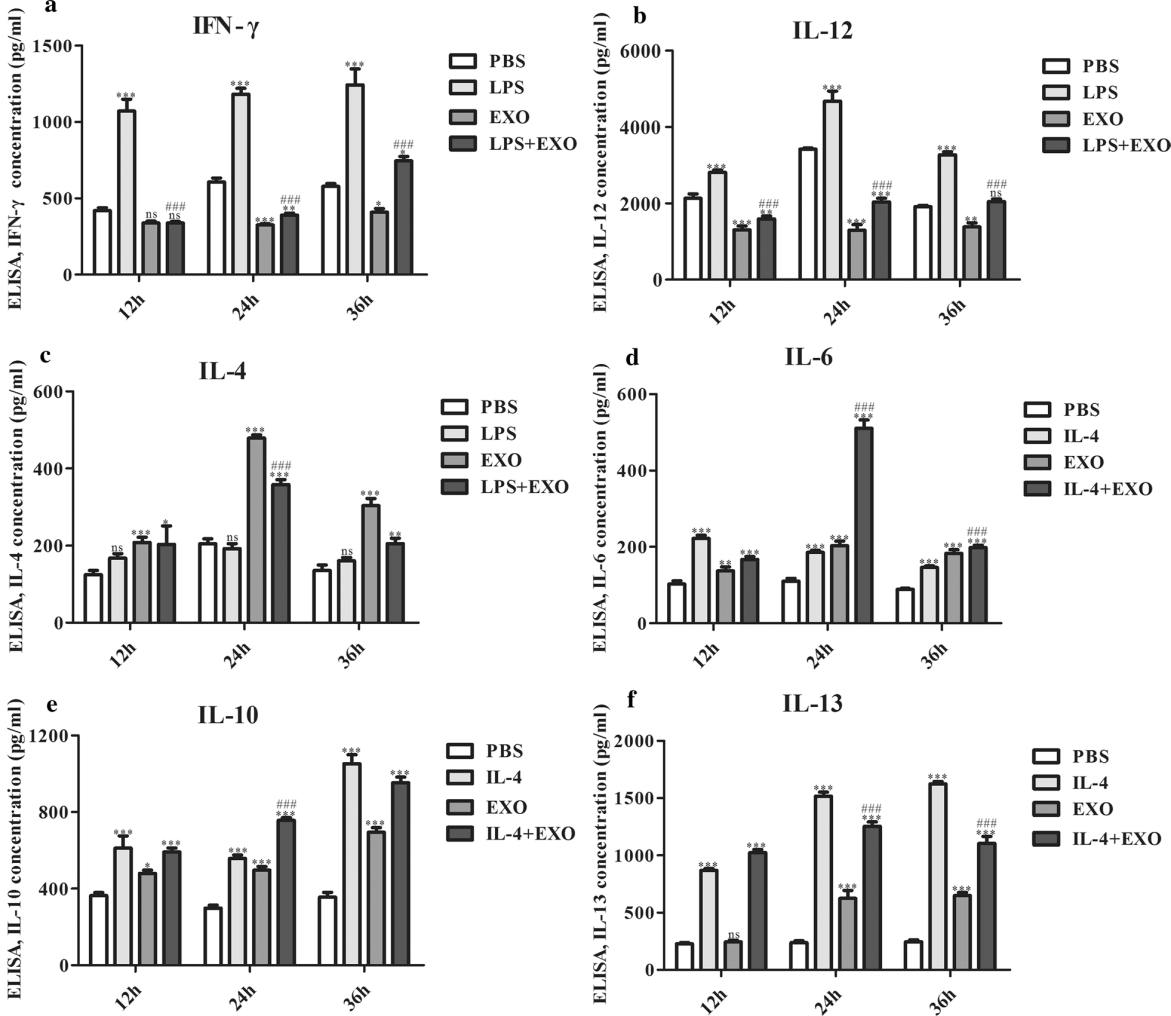
*T. pisiformis* cysticercus exosome-like vesicles stimulated production of M2 cytokines in RAW264.7 macrophages. LPS and IL-4 served as positive controls for M1 phenotype and M2 phenotype molecules, respectively. PBS served as the negative control for cytokine stimulation. **a**, **b** Induction of M1 markers in RAW264.7 macrophages treated with *T. pisiformis* cysticercus exosome-like vesicles. **c**-**f** Induction of M2 markers in RAW264.7 macrophages by *T. pisiformis* cysticercus exosome-like vesicles. Data for the final analysis are from three independent experiments and are expressed as the mean ± standard error (SE). *P < 0.05, **P < 0.01 and ***P < 0.001 were considered statistically significant compared to PBS-treated RAW264.7 macrophages. ^#^*P* < 0.05, ^##^*P* < 0.01 and ^###^*P* < 0.001 were considered statistically significant compared to LPS/IL-4 treated RAW264.7 macrophages (Additional file 6: Table S10)

To further validate the qPCR results, the supernatant from those groups were collected and used to detect the expression of IL-12, IFN-γ, IL-4, IL-6, IL-10 and IL-13. The results of ELISA assay showed that both the expression levels of IFN-γ and IL-12 at 12 h, 24 h and 36 h were significantly increased in macrophages after treatment with LPS, while the production of IFN-γ and IL-12 at 12 h and 36 h decreased significantly in macrophages stimulated with EXO or LPS + EXO compared with LPS treated cells (Fig. 8a, b). Macrophages treated with EXO obviously increased the level of IL-4 secretion at 12 h, 24 h and 36 h. However, cells treated with LPS + EXO produced remarkably lower levels of IL-4 after 24 h than did cells treated with EXO alone (Fig. 8c). The secretion levels of IL-6, IL-10 and IL-13 at 12 h and 24 h were significantly increased in macrophages treated with IL-4 (Fig. 8d–f), and the levels of IL-6 and IL-10 at 12 h and 36 h were also increased by treatment with EXO compared to the PBS control (Fig. 8d, e).The expression of IL-6 and IL-10 at 24 h were increased by treatment with IL-4 + EXO compared to the IL-4 control (Fig. 8d, e). The expression of IL-13 at 12 h had no obvious change after treatment with EXO. However, combination treatment with IL-4 + EXO tended to produce more IL-13 (*P* < 0.001) at 24 h and 36 h than did IL-4 treatment alone (Fig. 8f). Taken together, these data suggest that macrophages stimulated by exosome-like vesicles from *T. pisiformis* cysticercus produced mainly Th2 cytokines.

## Discussion

Exosomes, nano-sized endosome-derived membrane vesicles, play vital roles in intercellular communication [49]. An increasing body of studies has revealed exosomes as a ubiquitous molecular mechanism that can transfer bioactive molecules from pathogens to host cells in order to regulate host immune response or promote parasite survival [40]. In the present study, we isolated the exosome-like vesicles derived from *T. pisiformis* cysticercus, profiled their proteins and miRNAs, and further evaluated their immunomodulatory roles in RAW264.7 macrophages treated with exosome-like vesicles.

We identified seven enzymes involved in energy metabolism (glycolysis, gluconeogenesis and the tricarboxylic acid cycle). Among them, enolase, fructose 1, 6 bisphosphate aldolase and phosphoenolpyruvate carboxykinase are commonly observed in the context of parasite-derived exosome-like vesicles [30, 42]. It is well-known that, in addition to participating in the glycolysis and gluconeogenesis pathways, enolase can act as a plasminogen receptor as well, which prevents blood clots and facilitates parasite migration within hosts [36], suggesting that enolases packaged within exosome-like vesicles could be important for *T. pisiformis* cysticercus survival in hosts. Through proteomic analysis, we were able to identify multiple proteins related to exosome biogenesis, including Rabs (Rab-2A, Rab-4A, Rab-6A, Rab-10 and Rab-14), vesicular fusion protein, Vps4, transforming protein RhoA and Rab effectors otoferlin. Most of these molecules have been reported in exosome-like vesicles from *Echinococcus* and other flatworm parasites [27, 41]. In light of the fact that these proteins are typical molecules associated with the ESCRT-dependent pathway, we hypothesized that ESCRT is likely the major route involved in the formation of exosome-like vesicles and the sorting of cargo into exosome-like vesicles, consistent with a previous study of adult *Fasciola hepatica* [41]. Moreover, the proteomics analysis identified tegument-specific proteins from *T. pisiformis* cysticercus exosome-like vesicles, providing evidence for the possible roles of exosome-like vesicles in parasite survival through modification of the host immune response.

We identified 41 known miRNAs in *T. pisiformis* cysticercus exosome-like vesicles. The biological functions of the predicted targets of these miRNAs were associated mainly with immune system and signal transduction. Some of miRNAs could regulate innate immune response in inflammatory pathways through targeting signaling components. Previous studies suggested that let-7c-5p suppressed LPS-induced inflammation *via* targeting the DMP1-mediated NF-κB pathway [50]. miR-9 was induced by LPS in human monocytes and neutrophils, and increased miR-9 acts as a feedback control of the NF-κB dependent inflammatory response by inhibiting the expression of NFκB1 [51]. Moreover, miR-124 and miR-125 have been found to mediate inflammatory response and macrophage activation [52–54]. Thus, transferring these specific miRNAs to host cells might alter certain gene expression in macrophages. Furthermore, qPCR confirmed that eleven selected miRNAs, including seven known miRNAs (miR-71-5p, miR-10a-5p, miR-let-7-5p, miR-745-3p, miR-219-5p, miR-124-3p and miR-4989-3p) and four novel miRNAs (novel-mir-3, novel-mir-7, novel-mir-8 and novel-mir-11) existed in cysticerci and exosome-like vesicles of *T. pisiformis*. Compared to *T. pisiformis* cysticercus, exosomal novel-mir-7 had the highest relative abundance, followed by miR-let-7-5p, novel-mir-8, novel-mir-11 and miR-124, suggesting that these miRNAs from *T. pisiformis* cysticercus may be selectively encapsulated in exosome-like vesicles. However, little is known about their selective sorting mechanism and it remains to be elucidated in future studies.

The most significant finding of this study was that exosome-like vesicles from *T. pisiformis* cysticercus induced the macrophages polarization toward the M2 phenotype and produced a Th2-type immune response. When RAW264.7 macrophages were treated with *T. pisiformis* cysticercus exosome-like vesicles, the production of Arg-1, IL-4, IL-6, IL-10 and IL-13 was significantly increased. In contrast, the expression of iNOS, IFN-γ and IL-12 was significantly decreased, revealing that *T. pisiformis* cysticercus exosome-like vesicles participate in promoting macrophages to M2 polarization. There is evidence that injection of exosomes from the intestinal fluke *Echinostoma caproni* in BALB/c mice prime balanced Th2/Treg immune responses, which alleviates intestinal symptom severity in subsequent challenge infections and benefits parasite survival [55]. The immunomodulatory capacity of exosomes from the murine gastrointestinal nematode, *Heligmosomoides polygyrus*, has been demonstrated by suppressing innate type 2 lymphoid cell responses. Furthermore, *H. polygyrus* EVs have been shown to suppress the expression of IL1RL1/ST2, the IL-33 receptor, and type 2 innate lymphoid cell responses [56, 57]. Similarly, our previous studies showed that rabbits immunized with exosomes from *T. pisiformis* cysticercus displayed a higher production of IL-10, which results in decreasing significantly in worm reduction after challenging tapeworm eggs (our unpublished data). These studies suggest that exosome-like vesicles could play an important role in the host Th2-type immune response induced by *T. pisiformis* cysticercus.

In summary, although the presence of exosome-like vesicles has been demonstrated in several parasites, this is the first systemic study on exosome-like vesicles derived from ESP of *T. pisiformis* cysticercus in terms of morphology, size, content and immune regulation. The present work revealed that exosome-like vesicles participated in the process of parasite-host communication and the modulation of host Th2-type immune response induced by *T. pisiformis* cysticercus. The present investigation provides new insights into a deep understanding of molecular cargo in exosome-like vesicles of *T. pisiformis* cysticercus and the pathogenesis of exosome-like vesicle-mediated metacestodiasis.

## Conclusions

We successfully purified exosome-like vesicles from *T. pisiformis* cysticercus and profiled their protein and miRNA components, demonstrating the potential biological functions of exosome-like vesicles in the host immune response. Interestingly, the present study reveals the upregulation of molecules associated with the Th2-type immune response in RAW264.7 macrophages after stimulation with exosome-like vesicles from *T. pisiformis* cysticercus, which might facilitate survival of *T. pisiformis* cysticercus in rabbits. Further exploration of exosomal miRNA targets will be beneficial to elucidate the immunomodulatory mechanism and the important role of exosome-like vesicles in the interaction between the host and *T. pisiformis* cysticercus.

## Supplementary information

**Additional file 1: Table S1.** Primers for miRNAs qPCR validation. **Table S2.** Primers for qPCR of Th1/Th2 associated molecules.

**Additional file 2: Table S3.** Protein identification of exosome-like vesicles from *T. pisiformis* cysticercus. **Table S4.** GO analysis of exosomal proteins from *T. pisiformis* cysticercus.

**Additional file 3: Table S5.** miRNAs identified in exosome-like vesicles from *T. pisiformis* cysticercus. **Table S6.** miRNAs identified in *T. pisiformis* cysticercus. **Table S7.** Newly discovered miRNAs in exosome-like vesicles and *T. pisiformis* cysticercus.

**Additional file 4: Table S8.** The potential targets of *T. pisiformis* cysticercus exosomal miRNAs.

**Additional file 5: Table S9.** Results of statistical analyses of qPCR.

**Additional file 6: Table S10.** Results of statistical analyses of ELISA.

## Data Availability

Data supporting the conclusions of this article are included within the article and its additional file.

## Abbreviations

ACN: acetonitrile
Arg-1: arginase-1
DTT: dithiothreitol
ESP: excretory/secretory products
EVs: extracellular vesicles
FBS: fetal bovine serum
GO: Gene Ontology
HCD: high-energy collision dissociation
IAM: iodoacetamide
IFN-γ: interferon-γ
IL-4: interleukin-4
IL-6: interleukin-6
IL-10: interleukin-10
IL-13: interleukin-13
KEGG: Kyoto Encyclopedia of Genes and Genomes
LPS: lipopolysaccharide
M1: classically activated macrophages
M2: alternatively activated macrophages
MVBs: multi-vesicular bodies
NTA: nanoparticle tracking analysis
PAGE: polyacrylamide gel electrophoresis
PBS: phosphate-bufered saline
RPMI 1640: Roswell Park Memorial Institute 1640 culture media
SAg: soluble worm antigens
TEM: transmission electron microscope
